# HLA-DRB1 alleles in four Amerindian populations from Argentina and Paraguay

**DOI:** 10.1590/S1415-47572009000200002

**Published:** 2009-06-01

**Authors:** Maria L. Parolín, Francisco R. Carnese

**Affiliations:** Sección Antropología Biológica, Instituto de Ciencias Antropológicas, Facultad de Filosofía y Letras, Universidad de Buenos Aires, Buenos AiresArgentina

**Keywords:** HLA-DRB1, polymorphism, Amerindians, biological affinities

## Abstract

The major histocompatibility complex (MHC) is one of the biological systems of major polymorphisms. The study of HLA class II variability has allowed the identification of several alleles that are characteristic to Amerindian populations, and it is an excellent tool to define the relations and biological affinities among them. In this work, we analyzed the allelic distribution of the HLA-DRB1 class II locus in four Amerindian populations: Mapuche (n = 34) and Tehuelche (n = 23) from the Patagonian region of Argentina, and Wichi SV (n = 24) and Lengua (n = 17) from the Argentinean and Paraguayan Chaco regions, respectively. In all of these groups, relatively high frequencies of Amerindian HLA-DRB1 alleles were observed (DRB1*0403, DRB1*0407, DRB1*0411, DRB1*0417, DRB1*0802, DRB1*0901, DRB1*1402, DRB1*1406 and DRB1*1602). However, we also detected the presence of non-Amerindian variants in Mapuche (35%) and Tehuelche (22%). We compared our data with those obtained in six indigenous groups of the Argentinean Chaco region and in a sample from Buenos Aires City. The genetic distance dendrogram showed a clear-cut division between the Patagonian and Chaco populations, which formed two different clusters. In spite of their linguistic differences, it can be inferred that the biological affinities observed are in concordance with the geographic distributions and interethnic relations established among the groups studied.

## Introduction

In the human species, the major histocompatibility complex (MHC) includes the HLA (human leukocyte antigen) genes. It is located on the short arm of chromosome 6, between 6p21.31 and 6p21.33, and is characterized by a set of highly polymorphic genes. The function of the HLA genes is to present antigens to the T-cells. In addition, some of the class I genes are ligands for natural killer cell receptors. These histocompatibility molecules can be divided into two main groups: class I and class II molecules. Class I molecules are found in the HLA-A, HLA-B and HLA-C loci, and class II in the HLA-DR, HLA-DQ and HLA-DP loci. Both types of molecules are formed by heterodimers consisting of two chains, α and β, noncovalently bound to an extracellular domain, a transmembrane portion and a cytoplasmic tail (Fainboim *et al.*, 1999; Travers, 2000).

One of the characteristics of the HLA system is its high degree of polymorphism. The extensive gene distribution among populations has enabled the characterization of different ethnic groups and the development of several interesting studies in bioanthropology. In this context, the analysis of the HLA system variability is an excellent tool for evolutionary studies, migratory path reconstruction, the establishment of interethnic contacts, and for evaluating the relationships and biological affinity among different human groups. These studies, conducted at the serological level and, more recently, with the use of molecular techniques, have shown that Native Americans display a lower genetic diversity than other populations (Cerna *et al.*, 1993; Trachtenberg *et al.*, 1996; Fernández-Viña *et al.*, 1997; Salzano, 2002; Tsuneto *et al.*, 2003). In addition, it has been observed that certain allelic variants found in Amerindians are absent or have lower frequencies in other ethnic groups. In Native Americans, the alleles DRB1*0403,*0404, *0407, *0802, *0901, *1402 and *1602 are extensively distributed throughout America, whereas DRB1 *0411, *0417, *1406, and *1413 were detected mainly in indigenous populations of Latin America (Cerna *et al.*, 1993; Zhang *et al.*, 1993; Petzl-Erler *et al.*, 1997; Tsuneto *et al.*, 2003). A review on this subject was recently published (Salzano, 2002).

In Argentina, Pirosky *et al.* (1983), Vullo *et al.* (1984) and Haas *et al.* (1985) made serological determinations of the class I and class II HLA antigens in the Toba, Wichi and Mapuche groups, and molecular studies were performed mainly in some aboriginal populations of northern Argentina (Vullo *et al.*, 1992; Cerna *et al.*, 1993; Fernández-Viña *et al.*, 1997, Pando, 1998; Marcos, 2000) and also in a Mapuche group of Anecón Grande, province of Río Negro (Ginther *et al.*, 1993). However, there are no studies so far on the allelic distribution of HLA-DRB1 in Mapuche populations from the localities of Cerro Policía and Aguada Guzmán, in the Río Negro province, nor in the Tehuelche community of the province of Chubut. No studies were performed either in the Wichi groups of Santa Victoria Este, province of Salta, or in the Lengua community of the Paraguayan Chaco. Therefore, the aim of this study was: 1) to analyse the genetic diversity of the HLA-DRB1 locus in four Indian populations, two from the Argentine Patagonia region (Mapuche and Tehuelche) and the other two (Wichi and Lengua) from the Gran Chaco Region of Argentina and Paraguay, respectively; 2) to estimate the genetic distances among them; and 3) to compare the results obtained with those from other native groups of Argentina.

**Figure 1:**
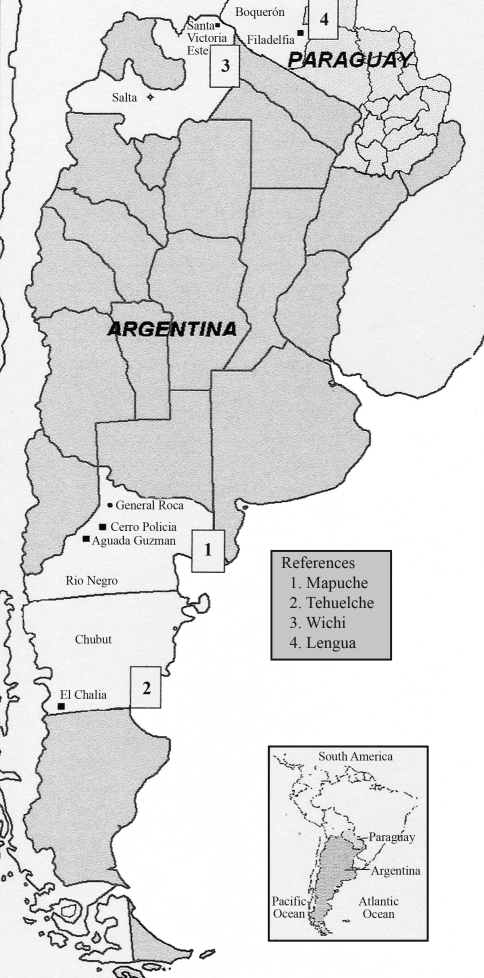
Map of Argentina and Paraguay showing the geographic locations of the Amerindian populations analysed in this report.

**Figure 2:**
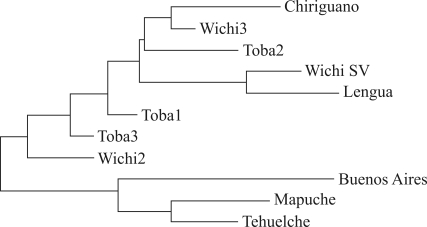
NJ dendrogram of the genetic distances (DA) among the analysed populations. For identifications and references, see Table 2.

## Materials and Methods

###  Populations studied

The Mataco or Wichi are much more numerous than the other Amerindian Chaco groups, their total population being estimated at 20,000 in 1986. A 1984 census revealed a total of 9,143 individuals in Salta, representing 51% of the Province's Indian groups. Besides agriculture, hunting and fishing, they are notable by their textile production (Acreche *et al.*, 1996). Their language, Mataco proper, is classified within the Mataco-Guaicuru linguistic stock (Loukotka, 1968; Greenberg, 1987). The sample was obtained from the community of Santa Victoria Este (SV), Department of Rivadavia, province of Salta (63° 42' W, 22° 17' S. See Figure 1)

The Lengua Indians live between 20° 30' and 24° S and 58° and 59° 50' W. They previously lived in Bolivia, and their language is classified as a stock of the Chaco division (Loukotka, 1968) or, according to the classification by Greenberg (1987), the Macro-Panoan branch of the Ge-Pano-Karib division. In the past, their life depended upon hunting, fishing and gathering. Currently, the main population is settled in communities near Filadelfia, Department of Boquerón, Paraguay (Figure 1), where they cultivate small gardens, plant crops, and have some livestock (Goicoechea *et al.*, 2001a). The individuals studied came from two communities, Misión Lengua, Barrio Obrero, Filadelfia, and Yalve Sanga, located 30 km south of Filadelfia.

The Mapuche speak a language classified within the Southern Andean division, the Araucanian group (Loukotka, 1968; Greenberg, 1987). They came from Chile and became established in Argentina since the beginning of the 17th century. Originally they mainly practiced agriculture, but nowadays they also extensively raise sheep and goats. They live in both rural and urban areas of the provinces of Rio Negro, Neuquén, Chubut, Santa Cruz, La Pampa and Buenos Aires. Their total population was estimated at 50,000 (Goicoechea *et al.*, 1996). The present sample was obtained from two localities: Aguada Guzmán (68° 57' W, 39° 30' S) and Cerro Policia (68° 37' W, 39° 10' S), province of Rio Negro (Figure 1).

The present-day Tehuelche are the remnants of a formerly quite large tribe that in historic times occupied the whole Patagonia, from the Rio Negro and its affluent, the Rio Limay, down to the Strait of Magellan and the isthmus connecting the Brunswick Peninsula with the continent. They were split into two main divisions, northern and southern, and those who live now are probably descendants of the southern group. Intermixture with non-Indians has occurred over the years (Valory, 1968). Their language is classified as Andean, Patagon stock (Loukotka, 1968; Greenberg, 1987). Material for the present study was collected at the village of El Chalia (71° W, 45° S), Department of Rio Senguer, province of Chubut.

###  Biological samples

A total of 98 individuals of Amerindian populations from the Patagonia Region [Mapuche (n = 34) and Tehuelche (n = 23)] and the Gran Chaco region of Argentina and Paraguay [Wichi SV (n = 24) and Lengua (n = 17)] were analysed (Figure 1).

The blood samples were obtained during the period from 1990 to 1995. Blood was collected into sterile tubes with anticoagulant and codified, anonymized and kept at -20 °C in the DNA Bank of the Biological Anthropological Section of the Anthropological Science Institute of Buenos Aires University. The donors of the samples were informed about the aim of the study and gave their consent to perform the research. The Wichi and Lengua samples were provided by Dr. J. Ferrer from Pennsylvania University, USA. DNA was obtained using the organic extraction method with phenol-chloroform and ethanol precipitation. The allelic variants of the HLA-DRB1 locus were determined using the PCR-SSOP (Polymerase Chain Reaction-Specific Oligonucleotide Probes) and PCR-SSP (Polymerase Chain Reaction-Sequence Specific Primers) molecular typing techniques. At first, sequence amplification of exon-2 was performed using primers that match homologous sequences for all the alleles of HLA-DRB1 loci. These products were scattered in a dot-blot shape on a positively charged nylon membrane, then denatured, fixed and later hybridized to several SSOP probes marked with digoxigenin. This first typing recognizes only the DRB1 family of alleles. The oligonucleotides used as probes or primers and the hybridization protocols were obtained from the XII International Histocompatibility Workshop (Bignon and Fernandez-Viña, 1997). For the determination of the allelic subtypes, the Orelup PCR-SSP kit was used according to the manufacturer's instructions.

###  Statistical analysis

The HLA-DRB1 gene frequencies were estimated using the maximum likelihood method (Excoffier and Slatkin, 1995). Hardy-Weinberg (HW) equilibrium was tested by calculating the exact p-values as proposed by Guo and Thompson (1992), using the ARLEQUIN program, version 2000 (Schneider *et al.*, 2000). Gene diversity and genetic distances (DA) were estimated using the method of Nei (1973, 1986) and Nei *et al.* (1983). Neighbour-joining dendrograms (Saitou and Nei, 1987) were elaborated using the DISPAN program (Ota, 1993).

## Results

Table 1 shows that in the four populations studied the alleles of Amerindian origin presented several variations. For example, while alleles DRB1*1402 and DRB1*0802 were detected in all groups analysed, allele DRB1*0403 was observed only in Wichi SV, Tehuelche and Lengua. The latter two groups shared the DRB1*1602 variant with the Mapuche Indians. However, in spite of the similarities detected, some of these variants presented different frequencies in the analysed groups. In turn, alleles DRB1*0411, DRB1*0417 and DRB1*1406 were detected only in the Gran Chaco region, while DRB1*0407 and DRB1*0901were detected exclusively in Patagonia.

Three out of the four populations were in HW equilibrium: Mapuche (p = 0.07), Tehuelche (p = 0.54) and Wichi SV (p = 0.11), whereas in Lengua there was a significant difference in the HWE (p = 0.02), with a pronounced deficiency of heterozygotes (Hobs = 0.36, Hesp = 0.55). In Mapuche and Tehuelche, 15 allelic variants were found, followed by Wichi SV and Lengua with 6 and 5 variants, respectively. The indigenous people of Patagonia exhibited a greater genetic diversity, probably due to the introgression of genes of European origin into their gene pool. In fact, these populations presented 35% and 22% of non-Amerindian genes, respectively (DRB1*01, DRB1*0301, DRB1*0405, DRB1*0701, DRB1*1001, DRB1*1101, DRB1*1102, DRB1*1104, DRB1*1301, DRB1*1302 and DRB1*1601, Table 1). In contrast, Wichi SV and Lengua showed 100% of Amerindian alleles and, therefore, the genetic diversity was much higher in Mapuche (H = 0.91) and Tehuelche (H = 0.85) than in Wichi SV (H = 0.75) and Lengua (H = 0.56). As usual in human populations, the intrapopulational variation was high and explained 86% of the total genetic variability, while the gene differentiation coefficient diversity (Gst') was 17%.

Our data were compared with those obtained in six Amerindian groups of the Gran Chaco region of Argentina and also with a sample of European origin from the city of Buenos Aires, to evaluate the effect of admixture (Tables 2  and 3). The results showed a pronounced divergence among these regions. In general, smaller genetic distances were observed among the Chaco Populations and also between the Mapuche and Tehuelche groups. Almost all populations showed clear-cut differences with regard to the sample of Buenos Aires. However, due to the high admixture of non-Amerindian genes of European origin, the Mapuche and Tehuelche presented much smaller or only minor genetic distances from the Buenos Aires group (Table 4). The genetic distance dendrogram (Figure 2) shows that the Patagonian-Buenos Aires populations are clustered together, separated from all the Chaco populations.

## Discussion

The results obtained show similarities and differences in the genetic composition of the Gran Chaco and Patagonia populations.

Comparing the data obtained with those of other American indigenous groups, we observed that allele DRB1*0802 was found in Central and South America at high frequencies in the Ijka (62%) of Colombia (Trachtenberg *et al.*, 1996) and in the Guarani (50%) and Kaingang (50%) of Brazil (Petzl-Erler, 1997; Tsuneto *et al.*, 2003). In the Indians from Argentina, this allele presented mean frequencies of 14% to 17% in the Wichi of Orán (Province of Salta), in the Toba that migrated to Rosario city (Province of Santa Fe) and in other Toba and Wichi groups from northern Argentina (Cerna *et al.*, 1993; Pando, 1998; Marcos, 2000). In our study, its prevalence was lower, with a variation range between 2% in the Wichi SV and 11% in the Tehuelche.

Allele DRB1*1402 was present at high frequencies in all four communities studied, similar to those observed in North American (Zuni = 32%, Tlingit = 52%) and in other South American (Kogui = 17%; Nukak = 65%) populations (Salzano, 2002).

Likewise, in the Wichi SV group, the frequency of allele DRB1*0403 (10%) was similar to those found in the Wayuu from Colombia (15%) and in the Yukpa from Venezuela (10%) (Petzl-Erler *et al.*, 1997), whereas the mean frequency of this allele has been reported to be 3% in Wichi, Toba and Chiriguano groups of the Argentinean Chaco region (Cerna *et al.*, 1993; Pando, 1998; Marcos, 2000).

The DRB1*0407 variant was not detected in the Wichi SV and Lengua, but presented high frequencies in the Mapuche (20%) and Tehuelche (32%) samples, in concordance with the variation range observed in North, Central and South America (19%-45%) (Salzano, 2002). In addition, allele DRB1*0901, which was detected only in the Mapuche (7%) and Tehuelche (9%), was also detected in the Quechua (18%) and in the Guarani (11%) of Peru and Brazil (Tsuneto *et al.*, 2003), and at much lower frequencies in indigenous groups of the Argentinean Chaco (Cerna *et al.*, 1993; Pando, 1998).

On the other hand, we detected the DRB1*1406 allele only in the Wichi SV (42%) and Lengua (65%) groups. These elevated frequencies were also observed in other populations of the Gran Chaco of Argentina: Wichi, Toba, Pilagá and Chiriguano, with a variation range going from 12% to 27% (Cerna *et al.*, 1993; Pando, 1998; Marcos, 2000). This allele was also found in some native Latin American groups such as the Terena (11%) of Brazil (Lazaro *et al.*, 1999) and, at lower frequencies (6%-10%), in the Mixe, Zapoteco and Mixteco of Mexico (Petzl-Erler *et al.*, 1997).

Allele DRB1*1602, which presented a variation range of 2% to 16% in Lengua, Mapuche and Tehuelche, was also detected by Cerna *et al.* (1993) in the Toba (2%), in the Chiriguano Indians (18%; Pando, 1998) of Orán, Province of Salta, Argentina, in the Mixe of Central America (31%), and in several South Amerindian populations such as the Wayuu (14%) and the Bari (40%) (Salzano, 2002).

It is worth mentioning that allele DRB1*0417 was detected for the first time in the Wichi and in the Toba by Cerna *et al.* (1993), Zhang *et al.* (1993) and in the Wichi studied by us. This allele was not observed in any other Amerindian populations and therefore we believe, in agreement with these authors, that this variant is original of the Argentinean Chaco Region.

In summary, based on the comparative analysis performed, we can conclude that most indigenous communities studied here share alleles with Amerindian populations of North, Central and South America. It should also be mentioned that the allelic variants detected here (DRB1*0403, *0405, *0802, *0901, *1402, *1406, *1602, DRB1*12 and DRB1*15) are frequent in Oriental populations (Grahovac *et al.*, 1998; Tokunaga *et al.*, 2001), mainly of northeast Asia, which is in concordance with the Asian origin of the American settlement.

On the other hand, the genetic distance dendrogram (Figure 2) shows that the Patagonian-Buenos Aires groups are clustered together and separated from all the Chaco populations. This topology is coherent with the geographic distribution and the interethnic relationships of the analysed groups. Thus, the strong biological affinities among the indigenous populations of Patagonia can be supported on the basis of the ethnic-historical information available. The contact between the Mapuche and Tehuelche populations began in the XVI century with the arrival of the Araucanian people in the Pampa and Patagonia regions. During this process, the Tehuelche incorporated the Mapuche language, as well as several cultural features. Thus, a major gene flow occurred through marriage, trade and political network (Mandrini, 1988; Nacuzzi, 1998). Likewise, these populations had intense contact with Spanish colonists ever since the XVIII century, mainly through trade relations and also upon conflictive and tense situations, when the seizing of captives made up the main axis for mating between natives and Europeans (Socolow, 1992). These facts could partly explain most of the genetic diversity observed in the Mapuche and Tehuelche Indians. In this sense, it is also known that in the communities of Aguada Guzmán and Cerro Policía there was a strong gene flow with non-Amerindians, due to the contacts established with people from urban centres such as the city of General Roca (Province of Río Negro) (Goicoechea *et al.*, 2000). Regarding the Tehuelche community of El Chalía, the presence of Europeans can be verified by the historical and demographic information available, given that they participated as colonists in the foundation of this settlement (Carnese *et al.*, 2002).

We did not have our own demographic data on the Chaco populations to allow us estimating the biological relationships among the groups studied. However, historical and anthropological information shows that the inhabitants of the Chaco kept extensive trade and cultural relationships that could encourage interethnic marriages with other Andean, Amazonian and Mesopotamian groups (Metraux, 1946; Dejean *et al.*, 2004). Therefore, the gene flow mechanism could explain the strong genetic affinities observed between the Wichi and Lengua groups. Nevertheless, the lower genetic diversity detected in these groups seems to contradict this explanation, because an interethnic gene flow leads to an increase in the intrapopulational genetic variability. Therefore, these results could be explained by a combined action of gene flow and genetic drift. This mechanism may have influenced mainly the Lengua group, as its lower biological diversity (H = 0.56) and its pronounced deficiency of heterozygotes seem to suggest. Previous data of STR markers obtained in the same populations also suggest that genetic drift could be the most important mechanism in the determination of the biological variability of Chaco populations (Catanesi *et al.*, 2006). However, the low diversity found in this group could also be due to the small sample size. It is known that natural selection has a certain effect on HLA loci, which can produce some distortion and lead to an erroneous assessment of the biological relations among populations. It should however be emphasized that our results are in concordance with those obtained in previous studies performed in the same population, using blood group determination and mitochondrial and nuclear DNA markers (Goicoechea *et al.*, 2001b; Carnese *et al.*, 2003; Catanesi *et al.*, 2006). This concordance, in turn, ratifies the usefulness of the HLA-DRB1 locus in genetic population studies.

## Figures and Tables

**Table 1:** HLA-DRB1 allele frequencies in the four populations studied.

Alleles		Populations			
HLA-DRB1		Mapuche (n = 34)	Tehuelche (n = 23)	Wichi SV (n = 24)	Lengua (n = 17)
DRB1* 01	01	0.059	0.022		
DRB1* 03	0301	0.059	0.022		
DRB1* 04	0403^		0.022	0.104	0.029
	0405	0.029			
	0407^	0.205	0.325		
	0411^			0.062	
	0417^			0.146	
DRB1* 07	0701	0.059	0.022		
DRB1* 08	0802^	0.089	0.108	0.021	0.059
DRB1* 09	0901^	0.074	0.087		
DRB1* 10	1001		0.022		
DRB1* 11	1101	0.015	0.022		
	1102	0.029	0.022		
	1104	0.044			
DRB1* 12	12		0.043		
DRB1* 13	1301	0.029			
	1302		0.022		
DRB1* 14	1402^	0.118	0.217	0.250	0.206
	1406^			0.417	0.647
DRB1* 15	15	0.015	0.022		
DRB1* 16	1601	0.015			
	1602^	0.161	0.022		0.059

n: number of samples; ^: most frequent alleles in Amerindian populations.

**Table 2:** Geographic and linguistic origins of the Argentinean populations that were compared with the Amerindian groups studied.

Population	n	Language	Locality	Reference
Chiriguano	56	Tupí-Guaraní	Orán (23° 08' S, 64° 20' W)	Pando, 1998
			Province of Salta	
Wichi (2)	19	Mataco Guaicuru	Orán (23° 08' S, 64° 20' W)	Pando, 1998
		Mataco Division	Province of Salta	
Wichi (3)	49	Mataco Guaicuru	Province of Formosa	Cerna *et al.*, 1993
		Mataco Division	(Ref: Mataco-Wichi)	
Toba (1)	135	Mataco Guaicuru	Province of Formosa	Cerna *et al.*, 1993
		Guaicuru Division	(Ref: Eastern-Toba)	
Toba (2)	19	Mataco Guaicuru	Province of Formosa	Cerna *et al.*, 1993
		Guaicuru Division	(Ref: Western-Toba-Pilaga)	
Toba (3)	86	Mataco Guaicuru	Rosario city (32° 57' S, 60° 39' W)	Marcos, 2000
		Guaicuru Division	Province of Santa Fe	
Buenos Aires	365	Spanish	Buenos Aires city (34° 38' S, 58° 28' W)	Marcos, 2000
			Province of Buenos Aires	

**Table 3:** HLA-DRB1 allele frequencies in six Amerindian groups of the Gran Chaco and a sample of Buenos Aires city.

Alleles		Populations						
HLA-DRB1		Chiriguano (n = 56)	Wichi (2) (n = 19)	Wichi (3) (n = 49)	Toba (1) (n = 135)	Toba (2) (n = 19)	Toba (3) (n = 86)	Buenos Aires (n = 365)
DRB1*01	01	0.009			0.016		0.058	0.082
DRB1*03	0301		0.026		0.026		0.006	0.110
	0302					0.030		
DRB1*04	0401	0.009					0.006	0.029
	0402							0.017
	0403^	0.018		0.021	0.034		0.052	0.014
	0404^	0.009	0.105	0.110	0.054	0.030	0.058	0.017
	0405				0.004		0.006	0.005
	0407^	0.053			0.062	0.061	0.081	0.019
	0408							0.010
	0409							0.005
	0410						0.006	0.005
	0411^	0.045	0.215	0.076	0.062	0.093	0.012	0.005
	0413							0.002
	0417^		0.080	0.122	0.090	0.061	0.041	
DRB1*07	0701	0.018	0.026	0.011	0.015	0.093	0.052	0.137
DRB1*08	0801						0.006	0.014
	0802^	0.107	0.110	0.183	0.223	0.126	0.203	0.027
	0803							0.005
	0804	0.026	0.026					0.010
	0806							0.002
	0807^	0.009						0.012
	0808							0.002
DRB1*09	0901^	0.018	0.053		0.011		0.017	0.019
DRB1*10	1001				0.004			0.012
DRB1*11	1101		0.026				0.012	0.063
	1102							0.007
	1103							0.010
	1104				0.037			0.031
DRB1*11	1106							0.002
	1108							0.002
	1115							0.002
DRB1*12	12	0.045						0.010
DRB1*13	1301			0.011	0.008		0.012	0.056
	1302	0.009					0.017	0.058
	1303				0.004			0.010
	1305							0.005
	1307							0.002
DRB1*14	1401				0.004		0.006	0.022
	1402^	0.214	0.186	0.264	0.106	0.093	0.145	0.014
	1404							0.002
	1406^	0.196	0.121	0.171	0.237	0.272	0.192	0.005
	1409		0.026					
DRB1*15	15	0.027		0.022	0.004	0.123	0.006	0.094
DRB1*16	1601							0.036
	1602^	0.179			0.023		0.006	0.007
	1604	0.009						
	1605							0.002

^: Most frequent alleles in Amerindian populations. For references, see Table 2.

**Table 4:** Genetic distances (DA) for ten populations of Argentina.

	Mapuche	Tehuelche	Wichi SV	Lengua	Chriguano	Wichi (2)	Wichi (3)	Toba (1)	Toba (2)	Toba (3)
Tehuelche	0.174									
Wichi SV	0.785	0.671								
Lengua	0.674	0.647	0.163							
Chiriguano	0.592	0.550	0.288	0.443						
Wichi (2)	0.357	0.307	0.339	0.228	0.330					
Wichi (3)	0.632	0.558	0.160	0.300	0.137	0.287				
Toba (1)	0.377	0.398	0.232	0.293	0.199	0.217	0.130			
Toba (2)	0.555	0.496	0.278	0.344	0.244	0.265	0.149	0.170		
Toba (3)	0.351	0.300	0.283	0.307	0.250	0.204	0.165	0.084	0.193	
Buenos Aires	0.341	0.427	0.815	0.809	0.593	0.517	0.649	0.485	0.567	0.387
